# Parent- and Intensivist-Reported Utility for Neonatal Genomic Testing

**DOI:** 10.1001/jamanetworkopen.2026.5689

**Published:** 2026-04-08

**Authors:** Katharine Press Callahan, K. Taylor Wild, Alexandra Heck, Laura Conlin, Matthew C. Dulik, David Munson, Chris Feudtner, Sara L. Reichert, Natalie Burrill, Ian Krantz, Nancy B. Spinner

**Affiliations:** 1 The Children’s Hospital of Philadelphia, Philadelphia, Pennsylvania; 2 Department of Medical Ethics and Health Policy, The Perelman School of Medicine at the University of Pennsylvania, Philadelphia; 3 Cohen Children’s Hospital, Long Island, New York

## Abstract

This survey study assesses intensivists’ and parents’ perceptions of the utility of rapid genomic testing for critically ill neonates.

## Introduction

Our understanding of the utility, or value, of rapid genomic testing for critically ill infants has been limited by indirect and incomplete metrics and omission of parental perspectives.^1,2^ We aimed to evaluate the utility of rapid genetic testing in critically ill neonates using comprehensive, parent- and intensivist-completed assessments that correct for prior shortcomings.

## Methods

This survey study was approved by the institutional review board at the Children’s Hospital of Philadelphia and followed the AAPOR reporting guideline. From January 2023 to January 2025, we distributed questionnaires (eFigures 1 and 2 in Supplement 1) to parents and intensivists 1 week after they received results from a large, rapid, genome-based panel, our hospital’s first-line genetic test for critically ill infants.^3^ We designed the questionnaire, in accordance with AAPOR recommendations, to expand on prior utility metrics by assessing perceived positive and negative outcomes rather than relying on changes of management (CoM) as proxies.^3^ Respondents were asked about the extent to which genetic information benefitted the patient overall on a Likert scale from 1 to 5, with a higher score indicating higher benefit. We analyzed results in Stata version 19.5 (StataCorp) to determine the proportion of patients who experienced outcomes. We used 2-sided *t* tests with a *P* = .05 threshold of significance for all comparisons. The survey began with a statement of informed consent.

## Results

We collected questionnaires from 143 parents and 71 intensivists regarding 154 infants (93 male [60%]; mean [SD] gestational age, 36.6 [3.0] weeks) (Table). Overall, intensivists (mean [SD] score, 4.0 [0.9] of 5) and parents (mean [SD] score, 4.1 [0.9] of 5) agreed that the genetic result benefited the patient. Most intensivists (57 intensivists [82%]) and most parents (139 parents [97%]) reported that testing had specific positive outcomes (Figure), most commonly confidence that one was not missing something and a clearer prognosis. Being valuable to a family was also a benefit frequently reported by intensivists, although an intensivist selecting this outcome was not associated with higher parent-reported benefit (selected: mean [SD] score, 4.0 [1.1] of 5; not selected: mean [SD] score, 4.1 [1.0] of 5; *P* = .59). Intensivists did not report expected reduction in morbidity, mortality, or length of stay for any patients despite reporting CoM for 31 patients (44%).

**Table.** Characteristics of Cohort

Category	Infants, No (%) (N = 154)
Length of stay, mean (SD), d	51.8 (57.9)
Gestational age, mean (SD), wk	36.6 (3.0)
Sex	
Female	61 (40)
Male	93 (60)
Unit of admission	
Cardiac intensive care unit	42 (27)
Neonatal intensive care unit	103 (67)
Other	3 (2)
Pediatric intensive care unit	6 (4)
Association of genetic variants	
No finding	105 (68)
Diagnostic	42 (28)
Incidental finding	7 (5)
Pathogenicity, No. of variants/total No.	
Pathogenic	30/53 (57)
Likely pathogenic	18/53 (34)
Variant of uncertain significance	5/53 (9)

**Figure.**
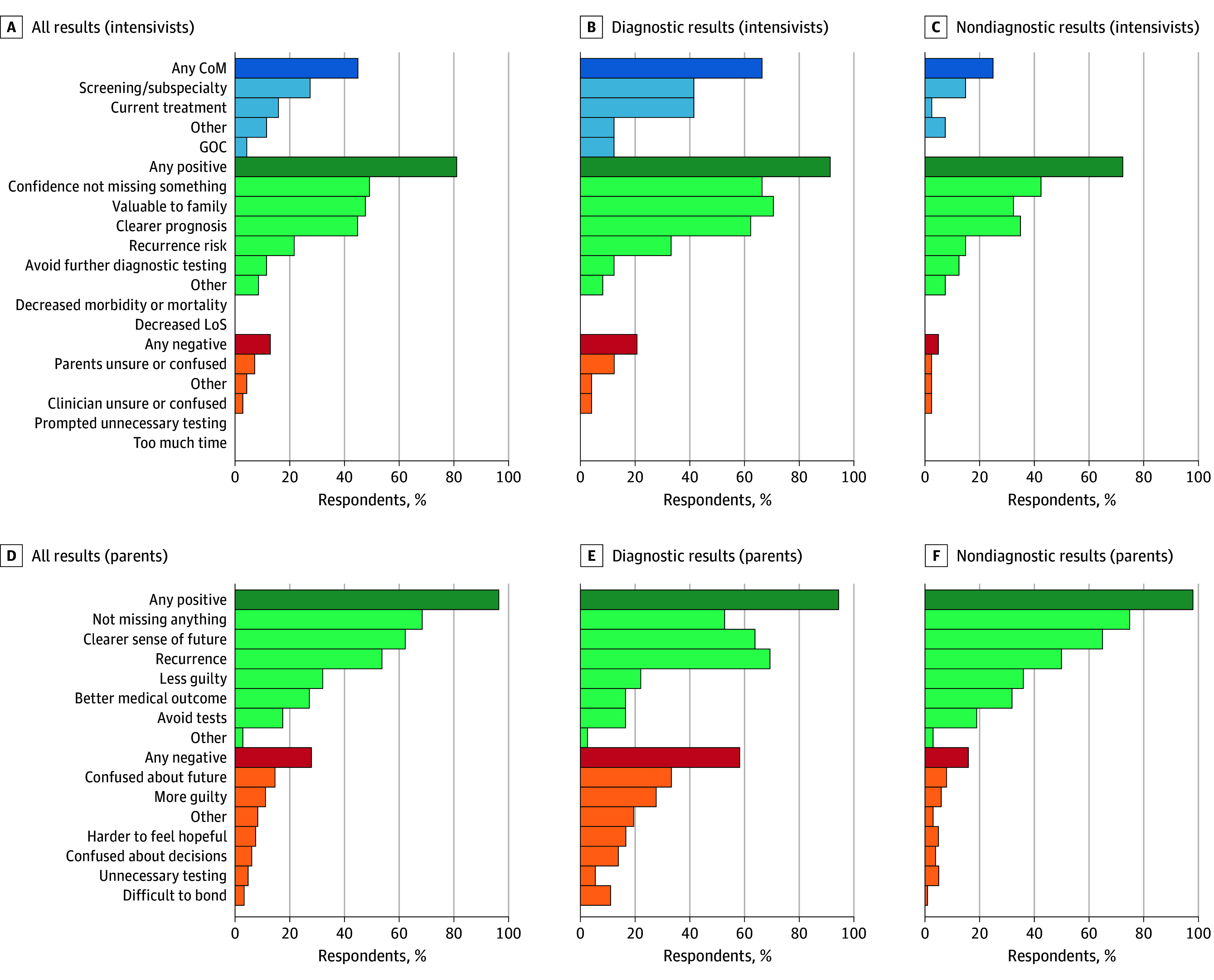
Bar Graph of Parent and Intensivist Reports of Changes in Management and Positive and Negative Outcomes Bars represent the percentage of respondents reporting each type of change of management (CoM; blue), positive outcome (green), or negative outcome (orange and red). GOC indicates goals of care; LoS, length of stay.

When asked about negative outcomes of the genetic test result, 9 intensivists (13%) and 41 parents (29%) reported yes to at least 1 item; most often it was parental uncertainty or confusion. Intensivists reported greater overall benefit if results were diagnostic (mean [SD] score, 4.4 [0.7] of 5), as compared with nondiagnostic (mean [SD] score, 3.9 [1.0] of 5) (*P* = .01), but parents reported less benefit (diagnostic: mean [SD] score, 3.8 [1.0] of 5; nondiagnostic: mean [SD] score, 4.1 [0.9] of 5; *P* = .03).

## Discussion

In this survey study, both intensivists and parents found rapid genetic testing to be useful, most often for informing prognosis and providing reassurance. In contrast with the conjecture of some researchers,^4,5^ genetic testing is rarely expected to decrease morbidity, mortality, or length of stay. Differences in testing modality are unlikely to explain discrepancies because the panel performed similarly to exome sequencing.^3^ More plausibly, discrepancies exist because our metric was more comprehensive and probed for a wider array of positive and negative outcomes beyond CoM. To our knowledge, no prior studies have directly assessed perceptions around short-term improvements in outcomes—although such gains have been presumed.

Our findings also suggest that family-centered utility has been given insufficient emphasis in the literature. If providing information that families value is a focus for intensivists, family-centered utility should be treated as a primary rather than secondary gain.^6^ Further, parents’ perspectives diverge from intensivists, indicating that nondiagnostic test results may be viewed as more beneficial than diagnostic ones, and all types of results have the potential for disutility, particularly confusion. Parents will need to report their perceptions of value directly, as intensivists’ proxy-reported assessments were, at least in this study, inaccurate.

Our study was conducted at a single center which limits generalizability and our ability to evaluate the many contextual factors that impact perceptions of utility. Further work should explore the ways in which counseling practices, sociodemographic forces, and timing of testing impact parents’ and clinicians’ appraisals. Nevertheless, we hope our findings may inform more complete and accurate measurement of utility.

## Notes

Supplement 1. eFigure 1. Clinician Utility Questionnaire eFigure 2. Parent Utility Questionnaire

Supplement 2. Data Sharing Statement
