# Supplementary material for: Parent- and Intensivist-Reported Utility for Neonatal Genomic Testing
Source: JAMA Netw Open. 2026 Apr 8;9(4):e265689. doi: 10.1001/jamanetworkopen.2026.5689 (PMC13063076; doi:10.1001/jamanetworkopen.2026.5689)
Supplement: Supplement 1. — eFigure 1. Clinician Utility Questionnaire eFigure 2. Parent Utility Questionnaire [file jamanetwopen-e265689-s001.pdf]

Supplemental Online Content

Callahan KP, Wild KT, Heck A, et al. Parent- and intensivist-reported utility for neonatal genomic testing. *JAMA Netw. Open*. 2026;9(4):e265689.
doi:10.1001/jamanetworkopen.2026.5689

eFigure 1. Clinician Utility Questionnaire

eFigure2. Parent Utility Questionnaire

This supplemental material has been provided by the authors to give readers additional information about their work.

© 2026 Callahan KP et al. *JAMA Network Open*.

eFigure 1. Clinician Utility Questionnaire

Confidential

Clinician Perceptions of Baby Eagle Testing

The goal of this brief questionnaire is to get your ideas about the effects the rapid targeted analysis of the genome (rTAG) test. This online questionnaire should take less than 10 minutes to complete. Participation is voluntary, and responses will be kept confidential to the degree permitted by the technology being used. Whether you participate will not impact your relationship with CHOP or be shared with their supervisor in a fashion that identifies you and will not adversely affect your employment. This survey involves minimal risk to you, including breach of confidentiality and minor discomfort answering questions about your patient's care. There are no direct benefits, however, information collected may impact society by helping to increase knowledge. You will be compensated with a $10 Amazon Gift Card for your time.

MRN

____

Patient's last name (during NICU admission):

____

Date of birth (MM/DD/YYYY):

____

Please select any changes in clinical management that have occurred or will occur because of this genetic result:

- ☐ Change in current treatment (that is, starting a new treatment or stopping or avoiding a treatment)
- ☐ Change in screening or subspecialty evaluation (similarly, starting, stopping, or avoiding screening or evaluation)
- ☐ Change in goals of care
- ☐ Other: ______
- ☐ No changes in clinical management

What was the change?

____

Please select any positive effects of the genetic result:

- ☐ Decreased morbidity or mortality
- ☐ Decreased length of stay
- ☐ Avoidance of unnecessary testing
- ☐ Clearer prognosis
- ☐ Clearer understanding of recurrence risk
- ☐ Increased my or the clinical team's confidence that we were not missing anything
- ☐ Was valuable to the family
- ☐ Other: ______
- ☐ No positive effects

What testing was avoided?

____

Is the decreased length of stay related to anticipated redirection of care?

- ○ No
- ○ Yes

Please select any negative effects of the genetic result:

- ☐ Led to unnecessary testing
- ☐ Took too much time away from other care or conversations
- ☐ Led me or the clinical team to feel unsure/confused
- ☐ Led family to feel unsure/confused
- ☐ Other: ______
- ☐ No negative effects

© 2026 Callahan KP et al. *JAMA Network Open*.

To what extent do you agree with the following statements:

	Strongly disagree	Disagree	Neutral	Agree	Strongly agree
I understood the genetic result completely.	○	○	○	○	○
The genetic result benefitted the patient or family.	○	○	○	○	○
If the result had been delayed 1 month, this would decrease its usefulness.	○	○	○	○	○

Please share any ideas about what could have made this genetic result more useful. ________________________________

© 2026 Callahan KP et al. *JAMA Network Open*.

eFigure 2. Parent Utility Questionnaire

Confidential

Parent Perceptions of Baby Eagle Testing

The goal of this brief questionnaire is to get your ideas about positive or negative effects of genetic testing for your child and family.

MRN	__________________
Child's last name in the hospital:	__________________
Date of birth (MM/DD/YYYY):	__________________
Please select any positive effects of the genetic result:	☐ Better medical outcome<br>☐ Avoiding medical tests my child didn't need<br>☐ Clearer sense of the future<br>☐ Better understanding of whether the same problem may affect future children<br>☐ Made me feel less guilty about my baby's condition<br>☐ Made me feel more confident that we were not missing anything<br>☐ Other: ______<br>☐ No positive effects
Please select any negative effects of the genetic result:	☐ Led to unnecessary medical testing<br>☐ Made me feel confused about what to expect in the future<br>☐ Made me feel confused about what decisions to make for my baby<br>☐ Made it feel difficult to bond with my baby<br>☐ Made me feel guilty about my baby's condition<br>☐ Made it harder to feel hopeful for my child<br>☐ Other: ______<br>☐ No negative effects

To what extent do you agree with the following statements:

	Strongly disagree	Disagree	Neutral	Agree	Strongly agree
I understood the genetic result completely.	○	○	○	○	○
The genetic result benefitted my child or family.	○	○	○	○	○
The genetic result improved my ability to cope with having a sick baby.	○	○	○	○	○

Please share any ideas about what could have made this genetic result more useful.

____

© 2026 Callahan KP et al. *JAMA Network Open*.
