# Supplementary material for: Parent- and Intensivist-Reported Utility for Neonatal Genomic Testing
Source: JAMA Netw Open. 2026 Apr 8;9(4):e265689. doi: 10.1001/jamanetworkopen.2026.5689 (PMC13063076; doi:10.1001/jamanetworkopen.2026.5689)
Supplement: Supplement 2. — Data Sharing Statement [file jamanetwopen-e265689-s002.pdf]

Data Sharing Statement

Callahan. Parent- and Intensivist-Reported Utility for Neonatal Genomic Testing. *JAMA Netw Open*. Published April 08, 2026. doi:10.1001/jamanetworkopen.2026.5689

Data
Data available: Yes
Data types: Deidentified participant data
How to access data: Available by email to corresponding author
When available: With publication

Supporting Documents
Document types: None

Additional Information
Who can access the data: Anyone requesting the data
Types of analyses: Any purpose
Mechanisms of data availability: Without investigator support
